# Speedy Spirochetes: An Unusually Rapid Progression of Multisystem Manifestations of Syphilis

**DOI:** 10.7759/cureus.107450

**Published:** 2026-04-21

**Authors:** Yasasvhinie Santharam, Hamza Choudhry, Alexis Fazio, Jane J Chang, Mackenzie Dyrda, Sandeep Grover

**Affiliations:** 1 Internal Medicine, University of Florida College of Medicine – Jacksonville, Jacksonville, USA; 2 Ophthalmology, University of Florida College of Medicine – Jacksonville, Jacksonville, USA

**Keywords:** anterior uveitis, gumma, maculopapular rash, ocular syphilis, syphilis

## Abstract

Syphilis is known as the great imitator due to its variety of clinical presentations. It is also known to have a widely variable timeline for each of its manifestations, although the causes of each of these differing timelines are not yet well established in the literature. The purpose of this case is to highlight a patient with rapidly progressive syphilis, including cutaneous and ocular manifestations, with a brief discussion of factors contributing to timelines and variability of syphilis presentations.

## Introduction

Syphilis is a sexually transmitted infection caused by the *Treponema* bacterial species, most often *Treponema pallidum *(TP). In the United States, the syphilis infection rate has increased over the last 20 years, and in 2023, there were more than 209,000 reported cases [1]. This was the highest number of reported cases since 1950 [1]. This disease is known as “the great imitator” as when untreated, it can progress to include renal, cardiac, gummatous, neurologic, ocular, and otic manifestations [2]. Though the number of cases has risen in the last few decades, the mortality rate is very low due to ease of treatment with penicillin [3]. It is thus very important to diagnose syphilis early to prevent further complications.

The progression of untreated syphilis is characterized by three stages. Primary syphilis is defined by a chancre, or painless ulcer, occurring at the spirochete inoculation site (usually genitalia), with accompanying lymphadenopathy [4]. 

Secondary syphilis occurs when the spirochete spreads throughout the body, causing systemic inflammation and infection. This stage is typically present 4-10 weeks after the development of the primary stage chancre [5]. Secondary syphilis could last as long as two years, with systemic symptoms eventually disappearing, but with the risk of progression to latent or tertiary stage syphilis if not treated appropriately.

Latent syphilis typically presents after secondary stage syphilis, prior to progression to tertiary stage syphilis, and by definition, is a time period where someone with syphilis does not have any systemic or ocular manifestations or symptoms of the disease. The latent phase has been documented to last anywhere from a few months to 30 years [5]. 

Tertiary stage syphilis, also known as late syphilis, has been documented to occur from 1 to 30 years after the primary infection with original manifestation of the localized painless chancre.

Other manifestations of this disease that can occur at *any *point in the disease course are ocular syphilis and neurosyphilis. The former can present as iritis or uveitis, while the latter can present as meningitis, tabes dorsalis, or general paresis.

While syphilis can have a wide array of presentations dependent on the individual, it is interesting to see in this case a very fast progression from the initial chancre to the development of a gumma seen in tertiary syphilis, with subsequent ocular manifestations.

## Case presentation

A 37-year-old male with a past medical history of chronic daily fentanyl and crack cocaine use presented to the Emergency Department (ED) from the custody of law enforcement with a diffuse rash across his upper back, chest, face, and arms. The patient first noticed a shiny, raw lesion on the side of his glans penis four months prior, which subsequently scabbed over, with the rash starting three months prior to admission, and an ulcerative lesion on his left knee two months prior to admission. The rash originated on his trunk and arms one day when he woke up and spread upward to his face. He attempted to treat it with over-the-counter hydrocortisone cream but did not notice improvement.

About six weeks prior to admission, he had presented to a local methadone clinic seeking treatment for chronic opioid use. He received STI testing as part of his intake evaluation, and his treponemal screening test was positive. He reported inconsistent condom use with a monogamous female sexual partner over the previous six months. However, he did not receive treatment at that time due to a reported childhood allergy to amoxicillin. The rash continued to progress to his face, and two weeks prior to admission, he began to notice left eyelid swelling, accompanied by watery and purulent discharge, photophobia, and blurred vision in the left eye without any symptoms in the right eye. He applied over-the-counter polyvinyl alcohol eye drops to the affected eye, but reported that this exacerbated his symptoms, and he eventually presented to the ED for further workup in the custody of law enforcement.

**Figure 1:**
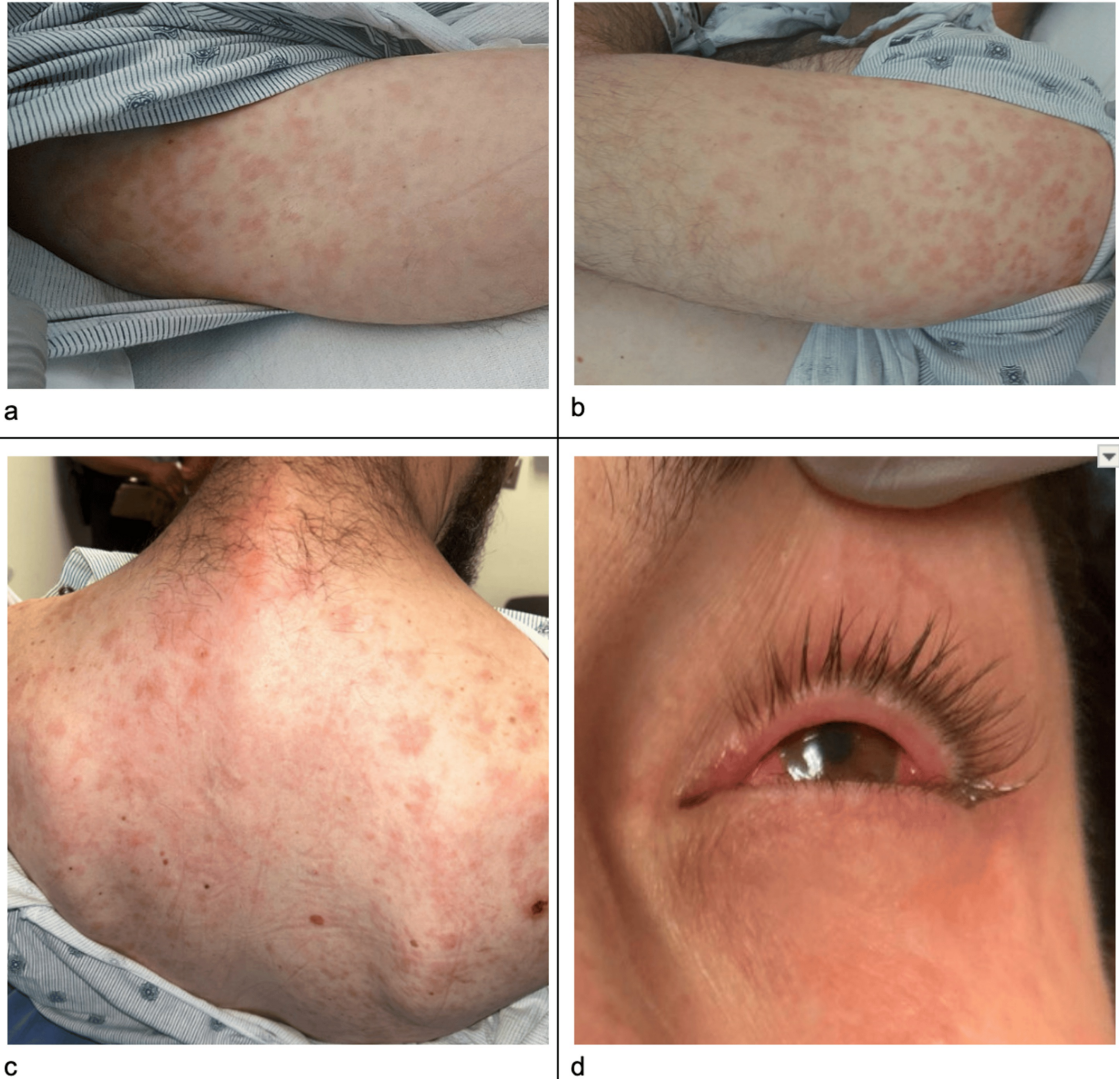
Macular erythematous rash covering the trunk, face, and bilateral upper extremities a) right upper extremity; b) left upper extremity; c) back; d) left eye and part of left cheek This macular (flat), erythematous rash without concurrent itching is consistent with that generally observed in the secondary stage of syphilis.

On admission, the patient was hemodynamically stable, with a blood pressure of 131/68 mmHg, heart rate of 76 bpm, respiratory rate of 18 breaths per minute, a temperature of 36.4 degrees Celsius, and oxygen saturation of 96% on room air. Physical examination revealed a diffuse, macular, erythematous rash covering the trunk, face, and bilateral upper extremities, with severe erythema and serous discharge from the left eye (Figure 1).

**Figure 2:**
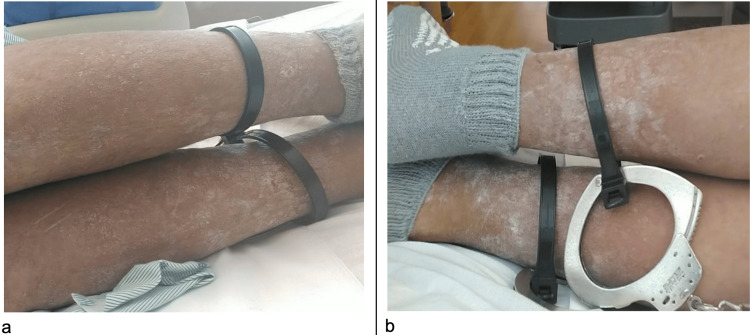
Scaly white rash with thickened nodular skin on lower extremities a) anterior shins; b) posterior shins This scaly, thickened, whitish-grey skin finding can also be seen in secondary stages of syphilis.

On the lower extremities, there was a flaky, scaly, white rash, and thickened, nodular skin over the bilateral ankles (Figure 2). The patient described the rash as non-painful and non-pruritic. No rashes were present on the palms or soles, and no mucosal lesions were present, with the patient denying having any of these previously.

**Figure 3:**
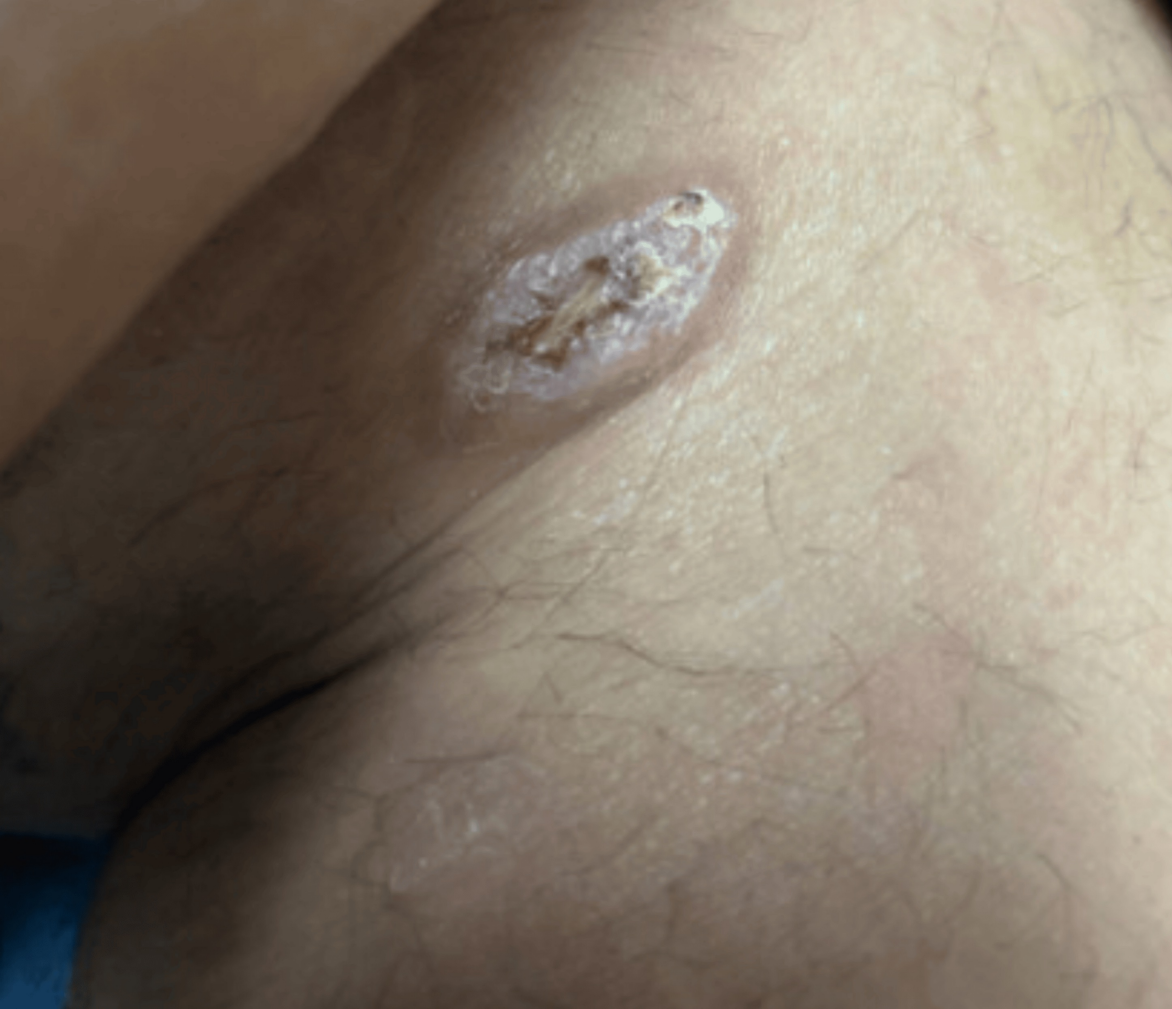
Gummatous, scabbed-over papule on left inner knee Gummas are soft granulomatous tissue growths with inflammatory cells causing tissue destruction, that are characteristic findings in tertiary syphilis. They consist of rubbery or firm nodules, or as in this case, an ulceration with a "punched out" necrotic center.

On further examination, he had bilateral inguinal lymphadenopathy and an ulcerative lesion on the medial aspect of his left knee, which he first noticed two months prior (Figure 3). Biopsy of the patient’s lesion was not performed because of presumed high pretest probability of a syphilitic gumma, though resolution of the wound with treatment of syphilis confirmed the diagnosis. This lesion was associated with progressive bilateral lower extremity edema that began at the feet and extended proximally past his knees.

**Figure 4:**
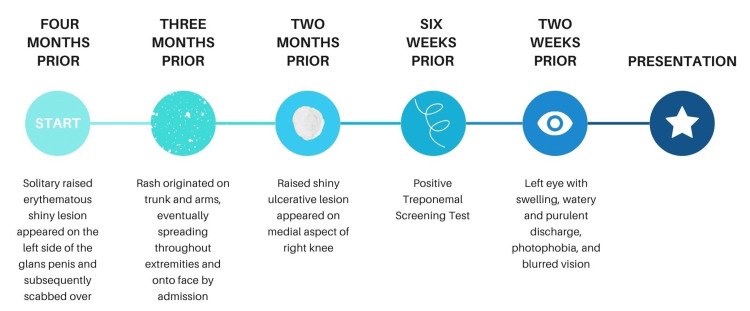
Symptom timeline

A clarification of the symptom timeline is noted in Figure 4.

Laboratory evaluation (Table 1) showed an unremarkable basic metabolic panel with no electrolyte abnormalities and normal creatinine levels, and a urinalysis was unremarkable and without proteinuria, though a complete blood count showed leukocytosis, indicating an infectious versus inflammatory process, as well as normocytic anemia with low iron levels and low iron saturation on the panel, indicating iron deficiency anemia. Given the lack of bleeding, his iron deficiency was likely in the setting of poor diet.

**Table 1:** Notable laboratory values on presentation

Laboratory Tests	Patient Laboratory Values	Normal Range Laboratory Values	Value Interpretation
Leukocyte count	9.42 x 10^9 cells/L	4-11 x 10^9 cells/L	Normal
Percent neutrophils	88.8%	34-73%	Elevated
Hemoglobin	11.3 g/dL	13.5-17.5 g/dL	Low
Hematocrit	35.8 %	40-54 %	Low
Mean corpuscular volume	88.5 fL	82.0-101.0 fL	Normal
Ferritin	72.5 ng/mL	30-400 ng/mL	Normal
Iron serum	43 μg/dL	32-159 μg/dL	Normal
Iron saturation	15%	20-55%	Low
Total iron binding capacity	283 μg/dL	261-390 μg/dL	Normal
Transferrin	223 mg/dL	200-360 mg/dL	Normal

Infectious workup yielded positive TP antibodies and a reactive RPR titer of 1:256, confirming syphilis infection. Tests for HIV 1/2 antigen/antibody, *Chlamydia trachomatis*, *Neisseria gonorrhoeae*, *Trichomonas vaginalis,* *Mycoplasma genitalium*, Hepatitis C antibody, Hepatitis B core antibody, Hepatitis B surface antigen, and Hepatitis A core antibody were all negative. Autoimmune workup revealed a positive ANA with nuclear envelope pattern at a titer of 1:80. Given the low titer level, further autoimmune workup (including complement levels, dsDNA, and ANCAs) was deferred.

Ophthalmology was consulted due to the patient’s complaints of left eye pain, blurred vision, and photophobia that had been present for two weeks in the setting of secondary stage syphilis diagnosis, denying prior occurrence of these symptoms. On the initial ophthalmic exam, the patient’s vision was 20/25 OD (Oculus Dexter i.e. right eye) and 20/100 OS (Oculus Sinister i.e. left eye). There was no reverse afferent pupillary defect, but the left eye did not constrict to light. Intraocular pressure was within normal limits in both eyes, although on the higher side of normal in the left eye. The left eye had 3+ limbal injection, compared to a white and quiet conjunctiva/sclera of the right eye. Limbal injection is typically associated with anterior segment inflammation, in cases like anterior uveitis, scleritis or conjunctivitis. The left eye notably had 3-4+ keratic precipitates and white/gray inflammatory cells on the corneal endothelium. Due to the corneal haze, an accurate evaluation of cells and flare in the anterior chamber could not be performed. Dilated fundus exam (DFE) showed 1+ optic disc edema with a poor view of the periphery OS, and a pink optic nerve OD with sharp margins. The patient was diagnosed with anterior uveitis OS.

At this time, given the patient’s constellation of findings including a nonspecific rash and anterior uveitis, a broad differential was considered in case there was another etiology for the symptoms and uveitis. There was an initial strong consideration of Behcet’s disease due to the diffuse nonspecific rash, fever, genital lesion, and uveitis, but it was ruled out due to lack of mucosal lesions, abdominal symptoms, or neurological symptoms, along with the timeline of the course and lack of pathergy with multiple IV lines. Additionally, given the low titer level of the ANA, lack of any joint inflammation, negative chlamydia/gonorrhea tests, and lack of genitourinary symptoms, reactive arthritis and other spondyloarthropathies were ruled out as well. As the patient had a diffuse macular rash, high RPR titer, positive TP antibodies, anterior uveitis, and inguinal lymphadenopathy, secondary syphilis was deemed the most likely etiology.

The state’s Department of Health was contacted prior to beginning treatment to confirm that the patient had never previously received treatment for syphilis. Infectious Disease was consulted for treatment recommendations. Despite a reported childhood allergy to amoxicillin, the patient tolerated a test dose without any adverse effects. He was then continued on intravenous penicillin G benzathine (12 million units daily for 14 days) and oral doxycycline (100 mg twice daily for 28 days). Topical treatment for uveitis in the left eye included prednisolone acetate 1% every six hours and timolol maleate 0.5% twice daily in the left eye only, and cyclopentolate 1% in both eyes.

On the next ophthalmic evaluation five days later, the best-corrected vision did not show any change in the left eye and DFE in the right eye showed a new finding of significant vitreous cells. The optic nerve swelling had resolved after initiation of penicillin G for syphilis. The patient was then subsequently seen in the ophthalmology clinic four weeks after presentation and visual acuity in the left eye had improved to 20/20 with resolution of uveitis. The topical treatment was then tapered off.

After treatment with penicillin G, ocular findings resolved, lower extremity edema had decreased, the erythematous rash significantly regressed, and the gummatous lesion disappeared, with the patient making a full recovery from all initial symptoms.

## Discussion

It is important to recognize the different stages of syphilis and be aware of the typical timeline for progression of the disease. The chancre seen in primary syphilis can manifest anywhere between 9 and 90 days after initial exposure [2]. Progression to secondary syphilis usually takes between 4 and 10 weeks after the initial chancre [2]. Tertiary syphilis can occur at any point afterwards, ranging from months to years after the initial infection [2].

In this patient, the three-month progression from initial chancre to the development of full-body macular rash, ocular syphilis, and a gummatous lesion was much more rapid than the usual course of the disease. Instances of gummas occurring within five months of presentation are very rare [6].

Per a literature review, there have been very few cases of rapidly progressing syphilis. There appears to be one case report that discusses a progression to tertiary syphilis within six months, and the patient in this case also happened to be immunocompromised with AIDS, leading to a discussion on possible faster disease progression in those who are immunocompromised [7]. However, our patient was not immunocompromised and thus does not fall into a similar category, prompting thought on why his disease progressed as such.

Additionally, it is known that while ocular syphilis can occur at any time, it is much more common to see it in the tertiary stage than the secondary stage [2]. Furthermore, posterior segment involvement in secondary-stage syphilis typically occurs in late-stage secondary syphilis [5]. However, this patient first developed primary syphilis only three months prior to the onset of his posterior segment involving ocular manifestations, even though the secondary syphilis stage can last up to a total of two years, further proving the rapid nature of his disease.

The clinical presentation of this case and variability of the timeline of progression begs the question as to why some patients have an earlier onset or wider variability of symptoms than others. It is important to note that TP and its subspecies do not produce cytotoxins or have any other identified virulence factors. Hence, the wider spectrum of symptoms cannot be ascribed to the virulence of the strain [8]. However, TP is known to evade immune response due to its adaptive abilities that include antigenic variability [8]. Another possible explanation could be the host’s delayed-type hypersensitivity (DTH) and humoral immunity to *Treponema pallidum*. As previously mentioned, progression of syphilis from the primary to secondary to latent and tertiary stages occurs in the absence of treatment, or essentially a failure to clear the infecting spirochetes. Several experimental studies in rabbits have shown that a strong DTH response is associated with faster clearance of the infecting organisms in a chancre, whereas a stronger humoral or cytotoxic T-cell response is associated with prolonged infection and more frequent progression to tertiary disease [9].

## Conclusions

In summary, this case highlights several manifestations of syphilis within a young adult male with no other comorbidities and interestingly points out the rapid rate at which symptoms can occur, even in those who are not immunocompromised. For physicians in the process of diagnosing a patient, it is a reminder to consider syphilis in several differentials, as it can present in so many ways across various body systems. Limitations to this broader generalization must of course be applied, as most cases of progressive syphilis will tend to occur within previously studied and noted timeframes, and it is rare that rapidly progressing cases occur, meaning physicians must still come up with a comprehensive set of differential diagnoses to not miss other disease processes that could cause similar presentations. However, when physicians advise folks about safe sex practices, particularly in areas where syphilis is endemic, it would benefit both patients and their partners to know the broad number of potential signs/symptoms to quickly seek treatment before progression of the disease. Looking forward, it is hoped that cases such as these inspire sexual health education curricula to include the broad spectrum of disease in syphilis and increase awareness of its acute and chronic manifestations, with the goal of using knowledge to encourage safe sex practices and decrease the incidence of disease.
